# Islander: a database of precisely mapped genomic islands in tRNA and tmRNA genes

**DOI:** 10.1093/nar/gku1072

**Published:** 2014-11-05

**Authors:** Corey M. Hudson, Britney Y. Lau, Kelly P. Williams

**Affiliations:** Sandia National Laboratories, Department of Systems Biology, Livermore, CA 94550, USA

## Abstract

Genomic islands are mobile DNAs that are major agents of bacterial and archaeal evolution. Integration into prokaryotic chromosomes usually occurs site-specifically at tRNA or tmRNA gene (together, tDNA) targets, catalyzed by tyrosine integrases. This splits the target gene, yet sequences within the island restore the disrupted gene; the regenerated target and its displaced fragment precisely mark the endpoints of the island. We applied this principle to search for islands in genomic DNA sequences. Our algorithm identifies tDNAs, finds fragments of those tDNAs in the same replicon and removes unlikely candidate islands through a series of filters. A search for islands in 2168 whole prokaryotic genomes produced 3919 candidates. The website Islander (recently moved to http://bioinformatics.sandia.gov/islander/) presents these precisely mapped candidate islands, the gene content and the island sequence. The algorithm further insists that each island encode an integrase, and attachment site sequence identity is carefully noted; therefore, the database also serves in the study of integrase site-specificity and its evolution.

## INTRODUCTION

Genomic islands are horizontally transferred DNA segments integrated into prokaryotic chromosomes. They came into especially sharp view when the second *Escherichia coli* genome (O157:H7) was sequenced and could be compared to the *E. coli* K-12 genome, revealing many large islands unique to O157:H7, some unique to K-12 and even alternative strain-specific islands found at the same chromosomal locus (1,2). Many such islands bear clues to their own mechanisms for their two most basic properties: integration and mobility. Like the prototypical genomic island, prophage lambda, many islands contain (i) an integration module including an integrase gene and (ii) the phage structural and regulatory genes that provide a ready hypothesis for the cross-bacterial mobility of the island, through phage particles. One major class of genomic islands can thus be identified as site-specifically integrated prophages. A second major class of islands displays an integration module together with genes for a Type IV secretion system (T4SS), suggesting that they move through conjugation pili like many plasmids; these are termed integrative conjugative elements (3,4).

Islands need not encode their own mobility or integration functions to be mobile and integrative. Islands with neither phage nor T4SS genes may nonetheless contain a mobilization signal, as do satellite phages and mobilizable plasmids, that accesses the mobility vehicles encoded by helper elements. Not all islands encode an integrase; some rely on the integrase-family host enzyme Xer, which is normally responsible for resolving dimeric chromosomes (5).

In addition to the island-selfish functions of mobility and integration, islands can also carry cargo genes benefitting the host bacterium, promoting for example virulence (as conveyed by the term pathogenicity island (6)), symbiosis or catabolic pathways, (7,8). Cargo-bearing islands are major agents of bacterial evolution.

A hypothetical pre-integration or post-excision circular form for genomic islands can be reconstructed from the genome sequence if the precise endpoints of the island can be determined. These circles have a ∼250 bp DNA segment termed *attP*, where integrase acts to promote recombination with a specific target site *attB* in the chromosome. For a large fraction of integrative islands (∼30–50%) (9,10), *attB* lies within a tRNA or tmRNA gene (together termed tDNAs). In these cases the island *attP* contains a fragment of the tDNA target, such that integration restores a functional tDNA despite disrupting the original tDNA. This leaves a bioinformatically detectable island signature in chromosomes: a tDNA and a tDNA fragment, with the island in between. Although some islands use integrases of the serine recombinase family, only those of the tyrosine recombinase family are known to target tDNAs; in what follows, we restrict the term integrase to members of the tyrosine recombinase family only.

Our algorithm Islander (11) searches for the above island signature, to find genomic islands that contain an integrase gene and target a tDNA *attB* and our website (http://bioinformatics.sandia.gov/islander) presents its results. Although this misses some islands, those that it finds are mapped with single-nucleotide precision. They are moreover putatively active, with an integrase gene and both *att* sites intact. Archaea, despite their many differences from bacteria, also carry genomic islands, many meeting our criteria.

Genomic islands are multigene mobile DNA units found in prokaryotic chromosomes (12). Some contain viral structural gene sets or conjugation gene sets that indicate their mode of mobility between cells. Chromosome site-specificity is determined by an integrase, overwhelmingly from the tyrosine recombinase family, whose gene is usually found on board the island. For approximately half of islands the integration target site is within a tRNA gene (9,10). Integrases of the serine recombinase family are not known to specify tRNA genes, and so we restrict the term ‘integrase’ in what follows to members of the tyrosine recombinase family only.

In addition to the selfish genes of mobility and integration, islands can carry cargo genes that benefit the host, contributing to phenotypes such as pathogenicity and metabolic repertoire (8). The target DNA genes specified by integrases have switched frequently during integrase evolution (10), facilitating the combinatorial accumulation of diverse islands in a given host genome. We describe our current algorithm for identifying tDNA-integrated, integrase-encoding genomic islands and present its results along with other such islands from the literature at the Islander Website.

## SEARCH STRATEGY

**Figure 1.**
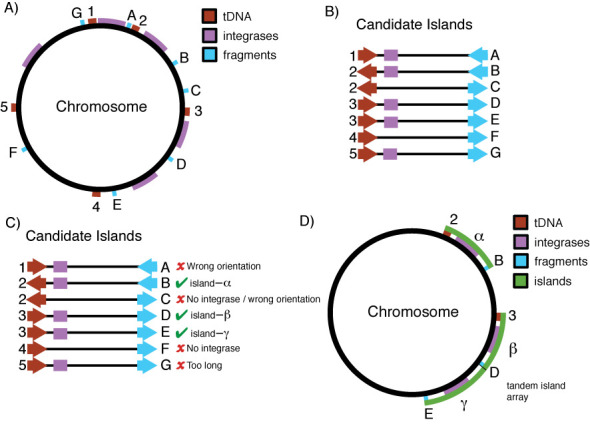
Islander algorithm. (**A** and **B**) Population Phase: tRNA and tmRNA genes (tDNAs), tDNA fragments and integrase genes are placed on the chromosome, and each interval between a tDNA and its cognate fragments is considered a candidate island. (**C**) Filtering Phase: Candidates pass through several filters, including tests for an integrase gene, correct fragment/tDNA orientation and length. (**D**) Resolution Phase: Multiple candidates at the same tDNA are resolved, identifying tandem arrays when each island in the array has its own tDNA fragment and integrase gene.

The Islander website collects t(m)RNA-targeted genomic islands, identified using the Islander.pl software package (http://bioinformatics.sandia.gov/software/). Changes from the previously described algorithm (13) include: six-frame translation and Pfam domain annotation; more precise identification of island integrases; and changing the Coding DNA Sequence (CDS) filter from using all proteins called at RefSeq to only those encoding Pfam domains (Figure 1).

The algorithm proceeds through each replicon as follows:

- *Find tDNAs*. tRNA and tmRNA genes are identified using tRNAscan-SE (tRNA), BRUCE (tmRNA), ARAGORN (both) and rFind.pl (two-piece tmRNA) (14–17). tFind.pl orchestrates implementation of these tools, corrects endpoints as necessary and sorts tDNAs with CAT anticodons into isoleucine, initiator and elongator methionine classes (18).
- *Find integrases, excluding Xer and integron subclasses*. The replicon is six-frame translated, with amino acid sequences extending from stop-codon to stop-codon. Genes for candidate integrases are identified using HMMER with the hidden Markov model (HMM) PF00589 from Pfam (19). The Xer proteins act at *dif* sites to resolve dimeric chromosomes. They can also act as integrases for islands but in these cases are not encoded within the island and specify integration into *dif* sites not tDNA sites. Xer proteins are excluded, using pfscan (20) with strict cutoffs on HAMAP (21) profiles for XerC, XerD, XerS and XerD-like subfamilies. Integron integrases mobilize cassettes within integrons, but not canonical islands. They were excluded using an HMM prepared from a MUSCLE (22) alignment of sequences from the ACLAME family Famint8 (23). Testing with all integrases from the ACLAME database, it was found that a threshold of 1.2e-24 was sufficient to segregate integron integrases from other integrases.
- *Find tDNA fragments*. Each tDNA is used as a query in a BLASTN search of its source replicon (parameters: task, blastn; gapopen, 0; gapextend, 2.5; word_size, 7). The hit and query gene define the endpoints of a candidate island, which is passed through several filters sequentially.
- *Integrase filter*. Candidate islands that do not contain or overlap an integrase open reading frame are rejected.
- *CDS filter*. Since true tDNAs and their island-split fragments should not overlap conserved portions of protein coding genes, Pfam-A domains are found among six-frame translated sequences and candidate tDNA fragments overlapping the domain portions of CDSs are excluded. As an exception, overlap of integrase domains is allowed, since some integrases (e.g. those from the viruses Mx8 and SSV) are known to extend across *attP* (24,25).
- *tDNA filter*. BLAST hits that fall within a known full-length tRNA gene are rejected.
- *Length filter*. Candidates shorter than 2 kb or longer than 200 kb are rejected. Some legitimate islands, e.g. the 611 kb symbiosis island of *Mesorhizobium loti* (26), are lost through this filter.
- *Internal tDNA fragment filter*. Integration splits off tDNA end fragments. Candidate islands where the tDNA fragment is internal to the full tDNA are therefore rejected. An exception was made at the 3′ end because certain islands have a small deletion in the tDNA fragment, 3 bp upstream of the discriminator position (27). To detect such damaged tDNA fragments, we allowed BLAST hits that extended only until this deletion site.
- *Configuration filter*. Integration displaces the tDNA fragment to one side. Candidate islands with a 5′ fragment downstream or with a 3′ fragment upstream of the tDNA are rejected.
- *Orientation filter*. Candidate islands with the tRNA gene in the opposite orientation from the fragment are rejected. This rejection step, coming late in the analysis, serves as a measure of the false positives among the candidates who should appear with equal frequency in each orientation.
- *Resolve*. Finally, cases where multiple remaining candidate islands share the same tDNA are resolved to single candidate islands, except when tandem arrays can be discerned where each member of the array has its own integrase and tDNA fragment.

## ISLANDER WEBSITE DESCRIPTION

The Islander Website was generated from 2031 whole bacterial genomes (that included 1640 plasmids and six phages) and 137 whole archaeal genomes (that included 75 plasmids), and from 1711 additional bacterial plasmids and 543 phages, and 44 additional archaeal plasmids and 38 archaeal viruses, downloaded from four directories at RefSeq (archaea, bacteria, plasmid, and viruses) in November 2012, rejecting eukaryotic viruses and plasmids. The Islander algorithm yielded 3927 unique islands, although all seven that were found in viruses and one in the smallest island-flagged plasmid were excluded after finding evidence that each was a false positive. Some statistics on the 3919 final genomic islands are shown in Table 1. In the majority of genomes at least one island was found; the highest number found in any genome was 19 (*Desulfovibrio magneticus* RS-1). As noted before (11), the tmRNA gene was more highly enriched among integration targets than any tRNA isoacceptor type.

**Table 1.** Statistics for Islander islands

Group	No.
Islander islands	3919
Islands that overlap RefSeq CDS	626
Overlapping RefSeq CDS called ‘hypothetical’	276
Tandems > = 2	125
Tandems > = 3	4
Whole genomes	2168
Genomes with at least one island	1302
Islands per genome with at least one (mean)	3.01
Islands with damage	148
Islands with 3′ tDNA fragment	3760
Islands with 5′ tDNA fragment	159
Islands with T-stem region *attP* site J^a^	1836
Islands with anticodon centered *attP* site A^a^	2083

^a^Attachment site crossover sites in tRNA genes appear to fall into three subsites, encoding the anticodon loop (A), the T loop, or the junction (J) between T and acceptor stems (). The latter two can be difficult to distinguish so we combine them here in J.

**Figure 2.**
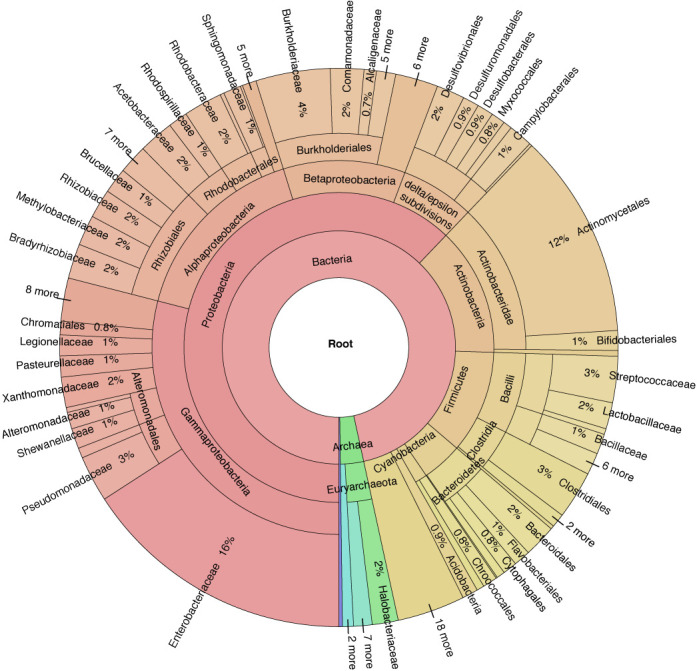
Taxonomic distribution of island prokaryotic hosts. The Krona metagenome visualizer (28) is used to display Islander islands by host, showing the preponderance of proteobacterial islands in the database. It has been modified to also serve as a navigation tool for the website.

Drop down menus become awkward with thousands of genomes and islands. We use a search text box and have also modified the javascript for an interactive metagenome taxonomy viewer, Krona (28), to enable navigation to individual island pages, while also providing a visual depiction of the phylogenetic distribution of islands in the database (Figure 2).

## COMPARISON WITH ORTHOGONAL METHODS

**Figure 3.**
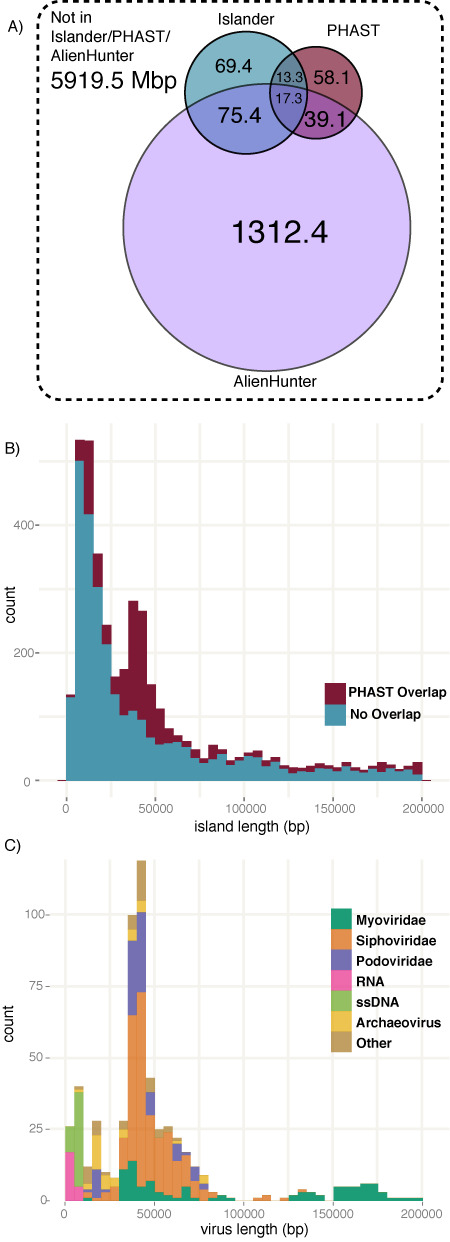
Island overlap. (**A**) DNA length overlap for the outputs from Islander, PHAST (prophage finder) and Alien_hunter (anomalous composition finder). (**B**) Size profile of Islander islands. For each island, overlap by at least 10% with PHAST regions was determined. The peak at 13.6 kbp has both PHAST and non-PHAST components, while that at 39.9 kbp is primarily from the PHAST-labeled islands. (**C**) Size profile of RefSeq prokaryotic viruses, broken down by high-level taxonomic group, matching both peaks of panel (B).

Our algorithm detects islands primarily by their target site, while other orthogonal methods look for phage-like genome content or for anomalous nucleotide composition. We evaluated the whole genomes in our study with PHAST (29) to identify prophage-like regions and with Alien_hunter (30) to find regions with biased composition. Figure 3A shows base-pair coverage of the genomes by Islander (2.34% of the total genome length), PHAST (1.70%) and Alien_hunter (19.3%), and their considerable overlaps. A key overlap is the 17.6% of the DNA of Islander islands that is covered by PHAST calls; the remaining PHAST regions may indicate prophages in sites other than tDNAs, or having lost an integrase gene or the tDNA fragment. Alien_hunter regions, despite their high genomic coverage, are enriched in Islander islands.

Figure 3B illustrates the island length distribution among Islander islands. There are distinct peaks centered at 13.6 kbp and 39.9 kbp, the latter mostly due to islands with PHAST overlap. These peaks are both found in the size profile of RefSeq prokaryotic viruses, which we show broken down by virus phylogeny in Figure 3C, with interesting trends. (Note that neither size profile necessarily represents the frequencies of islands or viruses in the natural world; each is biased by the selections researchers have made for genome sequencing.) The RefSeq profile matches best to the portion of the island profile with PHAST overlap (red segments in Figure 3B). The 40 kbp peak is enriched in *Podoviridae* and *Siphoviridae*. That the 40 kbp peak is negligible among non-PHAST islands (blue segments in Figure 3B) indicates that PHAST finds most of the Islander islands that are prophages, at least at this size range. Extrapolating, if nearly all self-mobilizing prophages among Islander islands are among the 1119 found by PHAST, that leaves ∼71% of Islander islands whose mobility must be explained by other means, perhaps either as PHAST-undetectable satellite phages or by conjugation. PHAST-overlapping islands are also enriched in the 13.6 kbp peak, but less so than in 40 kbp peak, and there may be a relative shift between PHAST and non-PHAST components within this peak. This peak is not explained by the single stranded DNA viruses populating that size range of Figure 3C, since none of them encode integrases.

## WEBSITE UPDATE

Since the website was last published (13), numbers have increased from 143 to 3919 islands, and from 106 to 2168 whole genomes treated. Our algorithm has changed as described above. We intend for Islander to be a gold standard repository of accurately mapped genomic islands, and are therefore currently combatting the few false positives that are mainly due to relaxing our CDS filter.

Our Islander naming convention takes the first letter of the genus name (excluding *Candidatus*) and the first two letters of the species name, adding a serial number to distinguish strains with the same three-letter nickname, then adds the island length in kbp and a single letter name for the integration site. As an example the 49 591 bp island in *E. coli* O157:H7 str. Sakai (Eco661) integrated into a tRNA-Ser gene is named Eco661_50S.

Additionally the updated website marks islands that are putative prophages overlapping with PHAST calls, reports all the integrases in the replicon, additional matching tDNA fragments, a gene list for the island and the island sequence.
